# Dashboard of Short-Term Postoperative Patient Outcomes for Anesthesiologists: Development and Preliminary Evaluation

**DOI:** 10.2196/47398

**Published:** 2023-09-19

**Authors:** Rama Syamala Sreepada, Ai Ching Chang, Nicholas C West, Jonath Sujan, Brendan Lai, Andrew K Poznikoff, Rebecca Munk, Norbert R Froese, James C Chen, Matthias Görges

**Affiliations:** 1 Department of Anesthesiology, Pharmacology and Therapeutics The University of British Columbia Vancouver, BC Canada; 2 Research Institute BC Children's Hospital Vancouver, BC Canada; 3 Department of Anesthesia BC Children's Hospital Vancouver, BC Canada; 4 Department of Anesthesiology Kelowna General Hospital Kelowna, BC Canada

**Keywords:** quality improvement, feedback, anesthesiologists, patient reported outcome measures, data display, user-centered design, surgical outcome, discharge, anesthesiology, postoperative care, registry, dashboard, interactive, practice, performance, patient outcome, mobile phone

## Abstract

**Background:**

Anesthesiologists require an understanding of their patients’ outcomes to evaluate their performance and improve their practice. Traditionally, anesthesiologists had limited information about their surgical outpatients’ outcomes due to minimal contact post discharge. Leveraging digital health innovations for analyzing personal and population outcomes may improve perioperative care. BC Children’s Hospital’s postoperative follow-up registry for outpatient surgeries collects short-term outcomes such as pain, nausea, and vomiting. Yet, these data were previously not available to anesthesiologists.

**Objective:**

This quality improvement study aimed to visualize postoperative outcome data to allow anesthesiologists to reflect on their care and compare their performance with their peers.

**Methods:**

The postoperative follow-up registry contains nurse-reported postoperative outcomes, including opioid and antiemetic administration in the postanesthetic care unit (PACU), and family-reported outcomes, including pain, nausea, and vomiting, within 24 hours post discharge. Dashboards were iteratively co-designed with 5 anesthesiologists, and a department-wide usability survey gathered anesthesiologists’ feedback on the dashboards, allowing further design improvements. A final dashboard version has been deployed, with data updated weekly.

**Results:**

The dashboard contains three sections: (1) 24-hour outcomes, (2) PACU outcomes, and (3) a practice profile containing individual anesthesiologist’s case mix, grouped by age groups, sex, and surgical service. At the time of evaluation, the dashboard included 24-hour data from 7877 cases collected from September 2020 to February 2023 and PACU data from 8716 cases collected from April 2021 to February 2023. The co-design process and usability evaluation indicated that anesthesiologists preferred simpler designs for data summaries but also required the ability to explore details of specific outcomes and cases if needed. Anesthesiologists considered security and confidentiality to be key features of the design and most deemed the dashboard information useful and potentially beneficial for their practice.

**Conclusions:**

We designed and deployed a dynamic, personalized dashboard for anesthesiologists to review their outpatients’ short-term postoperative outcomes. This dashboard facilitates personal reflection on individual practice in the context of peer and departmental performance and, hence, the opportunity to evaluate iterative practice changes. Further work is required to establish their effect on improving individual and department performance and patient outcomes.

## Introduction

Anesthesiologists benefit from receiving feedback on their patients’ outcomes and can use it to evaluate and improve their practice. The perioperative period is a data-rich environment with the potential for innovation through digital health tools and predictive analytics. Data-driven performance feedback can improve perioperative practice and outcomes [1,2], including antibiotic administration [3,4], drug costs [5], operating room booking efficiency [6], and temperature monitoring compliance [7]. Feedback is most effective when it is locally relevant and derived from a credible source [8]. Personalized feedback, provided in (near) real time [3,9], is more effective than retrospective and department-wide feedback [8]. However, evaluating postoperative care metrics can be challenging, particularly for day-case procedures: access to this information is often only available from fragmented data sources, data are difficult to access, or are presented in user-unfriendly formats.

In anesthesia, practitioners commonly work in isolation (1 anesthesiologist per patient) and do not often have a chance to compare variations in individual practitioners' anesthetic techniques and outcomes unless required by a critical or near-miss event. While providing care, anesthesiologists make multiple decisions influencing pain and nausea outcomes [10]. Longitudinal follow-up of patients is often limited to inpatients; in high turnover pediatric operating rooms, an anesthesiologist may not have time to check in on a recovering patient before they are discharged home, and postanesthetic care unit (PACU) nursing does not routinely inform an anesthesiologist when administering ordered doses of analgesic or antiemetic rescue medications. Hence, an anesthesiologist may not know how their patients are faring in the postoperative period. This suggests that a system to collate and visualize data for comparative feedback may allow anesthesiologists to fine-tune their decisions, optimizing pain and nausea outcomes.

As part of an organizational quality initiative, a postoperative follow-up (POFU) registry has been established at BC Children’s Hospital, a tertiary pediatric hospital in Vancouver, British Columbia, Canada. Its purpose is to understand the recovery experience of day-case surgical patients and to facilitate associated quality improvement endeavors. The POFU registry is maintained by PACU clerks and nurses, who record day-surgery patient information and short-term outcomes from PACU and then follow-up with families via telephone to gather patient-reported outcomes at 24 hours post discharge. These data are recorded using the Research Electronic Data Capture (REDCap) web application (Vanderbilt University) [11,12] hosted locally. Each patient’s contact information, demographics, clinician information, and procedure characteristics are entered using operating room scheduling system data. Validation checks are enabled for each field to ensure minimal artifacts; the data steward runs further reports and alerts to optimize data quality. Family-reported 24-hour outcomes (including postoperative pain, nausea, and vomiting) are collected by nurse telephone follow-up beginning in September 2020. PACU opioid and antiemetic administration, indicating early treatment of postoperative pain and nausea, have also been captured since April 2021.

This study’s primary aim was to turn these outcome data into accessible and actionable information by creating dashboards, which allow the anesthesiologists to evaluate their patients’ postoperative outcomes and reflect on their care. We also aimed to evaluate anesthesiologists’ perception of the dashboard. By providing anesthesiologists with single-point access to these outcome data, allowing them to reflect on their care, drill down on details, and compare their performance to their peers and to the department aggregate in a time-efficient and user-friendly way, we aim to facilitate ongoing individual and departmental practices of improvement.

## Methods

### Study Design

We initially conducted a literature search of previous dashboard designs and partnered with a group of anesthesiologists in our department to identify their information needs and to co-design dashboards using an iterative development process. We then designed a dashboard architecture based on the POFU registry data; this incorporated key security features required to meet institutional policies and the confidentiality requirements of our anesthesiologists. We deployed the final design to the anesthesia department and conducted a preliminary usability evaluation.

### Ethical Considerations

The University of British Columbia and Children’s and Women’s Health Centre of British Columbia Research Ethics Board determined this work to be established under a quality improvement or quality assurance (QIQA) framework (reviewed June 29, 2021), for which they do not require ethical review, in accordance with Article 2.5 of the Canadian Tri-Council Policy Statement. Data used in this study were obtained from the POFU registry, also established under a QIQA framework. This paper adheres to the Standards for Quality Improvement Reporting Excellence (version 2.0) guidelines [13].

### Registry Data and Dashboard Architecture

#### Exploratory Data Analysis, Cleaning, and Processing

We performed an exploratory analysis in Python (Python Software Foundation) to confirm that the collected registry data were clean: variables were evaluated for any out-of-range values; anesthesiologist codes (departmental QIQA identifiers) and procedure codes were verified against lists of valid entries. Age, sex, and procedure group of PACU and missing 24-hour outcome data were compared to examine any significant differences in the underlying data. Cases with missing 24-hour outcomes or unanswered phone calls were excluded from the 24-hour outcome analysis but not censured from the PACU outcome analysis.

Nausea and vomiting data were collected using a 4-point scale (none, mild, moderate, or severe). Pain scores in PACU are collected using developmentally appropriate observational or self-report tools, and 24-hour pain scores are obtained by proxy from a parent or caregiver using a 0-10 numeric rating scale [14]. We then assigned the same 4-point labels to the numeric pain ratings obtained: 0=none, 1-3=mild, 4-6=moderate, and 7-10=severe. For most dashboard purposes, we dichotomized variables into none or mild versus moderate or severe.

#### Dashboard Architecture and Data Sources

**Figure 1:**
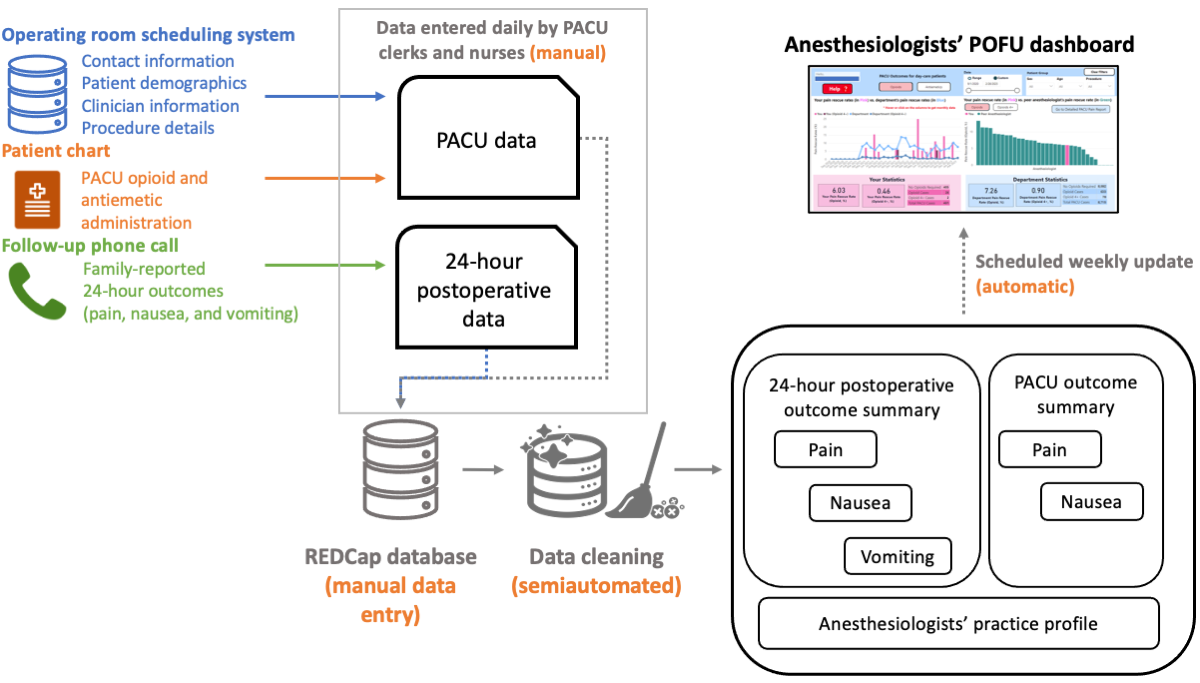
The overview of the data flow from POFU data collection for PACU and 24-hour phone call into the POFU dashboard. PACU: postanesthetic care unit; POFU: postoperative follow-up.

Our dashboard design aimed to provide a self-service platform for anesthesiologists to confidentially review their performance through postoperative (PACU and 24-hour) outcomes and anonymized peer comparison (Figure 1). The dashboards were developed using business analytics software Power BI Report Server (Microsoft), hosted by the BC Children’s Hospital Research Institute (BCCHR). Power BI uses Active Directory Federation Services (Microsoft) to leverage hospital and research institute single sign-on and can, in principle, load data from REDCap, network drives, or other systems via application programming interfaces. At our institution, it is maintained by the BCCHR Data Management team, who manages access to team spaces in the server and determines access and usage policies.

Deidentified POFU registry data are downloaded weekly by the data steward as a comma separated values file and stored on a BCCHR secure data drive, that is, instead of employing an application programming interface token and directly linking these systems. BCCHR data management policies imposed the need for this workaround, as the POFU REDCap registry contains personal health information, parental contact information, and anesthesiologist and surgeon identifiers.

The dashboards require 3 files: the POFU weekly data export (excluding any identifying data), a Power BI username-QIQA code mapping file (see dashboard security and confidentiality), and an auxiliary file containing outcome definitions (severity level and numeric score mapping), procedure definitions (procedure code and group mapping), and age categories (National Institute of Child Health and Human Development pediatric age categories) [15].

The POFU registry and auxiliary files are set as automated data sources in Power BI, automatically refreshing the dashboard whenever files are updated. In contrast, the mapping file was added manually as a data source by the POFU data steward and cannot be downloaded by any other team member. Power BI pulls new data from these sources every Monday morning via a scheduled refresh.

The dashboard is available to anesthesiologists through a web browser and is typically accessed on a desktop or laptop computer; it can be viewed on a tablet or a smartphone, though it has not been optimized for use in this way.

#### Dashboard Security and Confidentiality

Comparative data are required to contextualize practice patterns for postoperative outcomes, but our team of co-design anesthesiologists insisted that the security and confidentiality of both patient and provider data were imperative for departmental support: patient confidentiality is maintained by excluding identifiers from the data accessed by the dashboard; provider confidentiality is maintained through the use of departmental QIQA codes for anesthesiologists; see dashboard architecture and data sources. Data for the active anesthesiologist (ie, the user accessing the dashboard) and the department’s aggregate performance are presented together, but access to the underlying data is restricted, preventing access to other anesthesiologists’ data.

Power BI’s row-level security feature filters the nonaggregated case data to include only data for cases the active anesthesiologist performed; hence, each user can only see their own cases. This is achieved via the Power BI username-QIQA code mapping. Access to this file is restricted to the POFU data steward, maintaining strict confidentiality of the anesthesiologists’ identifiers.

### Dashboard Design and Evaluation

#### Iterative Co-Design Sessions

**Figure 2:**
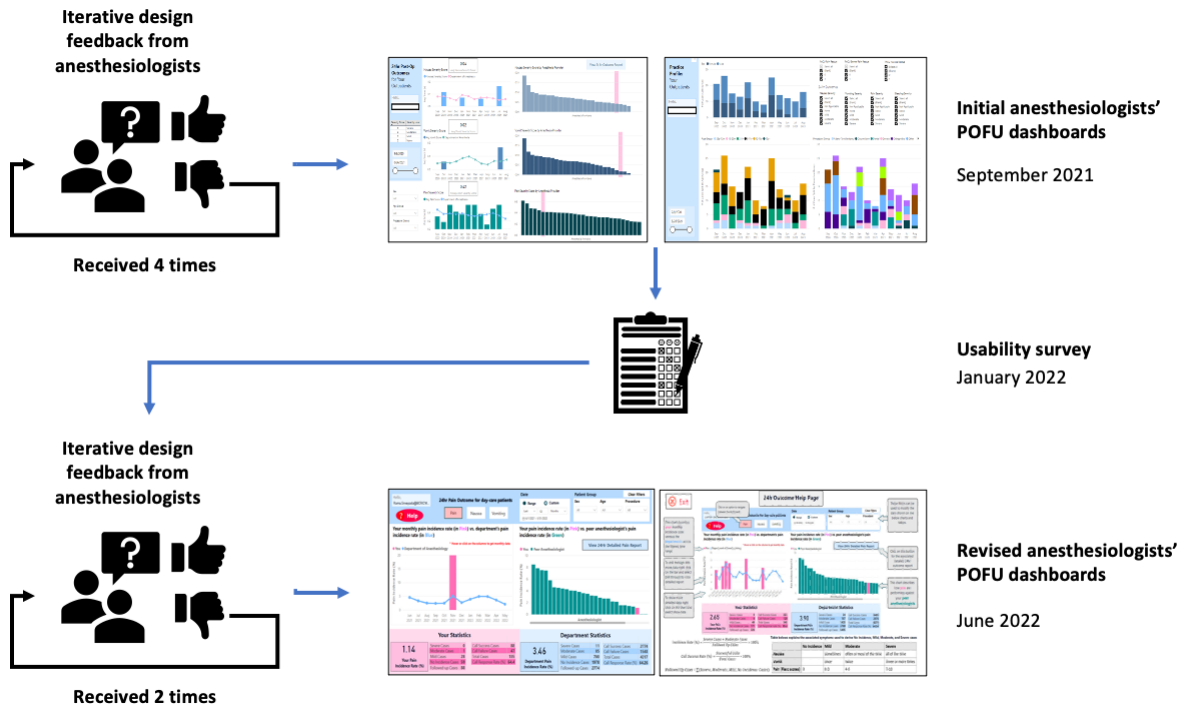
Iterative co-design process for the POFU dashboard. POFU: postoperative follow-up.

We performed a brief literature search of papers published in 2015-2021 to understand existing anesthesia dashboards; keywords included *dashboard, run charts, personalized feedback, anesthesia feedback,* and *surgery feedback* [7,16-23]. Our preliminary visualization designs were based on the ideas drawn from this literature search. Guided by the recommendation to incorporate users’ feedback into the dashboard development [16,19,23-26], we developed our system using a participatory design approach: we discussed design ideas with 5 anesthesiologists using iterative feedback to improve visualization. We adopted a convenience approach to selecting the design team, which consisted of the POFU clinical lead and other anesthesiologists who expressed an interest in contributing to the design process. Designs were demonstrated through screenshots and dynamic working prototypes during 6 feedback sessions; the visualizations were iteratively refined based on anesthesiologists’ comments and observing their use of the prototype dashboards (Figure 2). Each iterative feedback meeting on Zoom (Zoom Video Communications Inc) lasted approximately 1 hour.

The initial dashboard prototypes showed a given anesthesiologist's average 24-hour postoperative pain, nausea, and vomiting scores and compared them with the department’s average. Outcome scale distributions were also plotted. A second design added PACU outcomes (opioid and antiemetic administration). These were shown to the anesthesiologists, and the visualizations were iteratively refined to arrive at the final set of dashboards.

After the iterative development process, dashboards were deployed to the department in September 2021; the work was presented at departmental rounds, and anesthesiologists were emailed links to the tool with instructions and a contact email for further information. Subsequently, 2 further feedback sessions were conducted with our collaborating anesthesiologists to refine the final dashboard designs.

#### Usability Survey

To gather clinicians’ feedback on the initial version of the dashboard, a usability survey was distributed to all department anesthesiologists in January 2022 after this an initial version of the dashboard had been available for 4 months. It consisted of 21 required and 18 optional follow-up questions on aspects such as frequency of use (including reason, if infrequent), ease of use (including clarity of information displayed and usefulness of the instructions), content (helpfulness and suggestions for other functionality), impact on practice, and overall opinion of the dashboard (Multimedia Appendix 1).

Based on the feedback received from the usability survey, the dashboards were redesigned and redeployed to the department in June 2022; the dashboards were again presented at department rounds, and anesthesiologist superusers were identified to provide training and peer support.

### Dashboard Component Design

#### Overview

There are three dashboard components: (1) 24-hour outcome summary, (2) PACU outcome summary, and (3) anesthesiologists’ practice profile.

#### 24-Hour Outcome Summary

**Figure 3:**
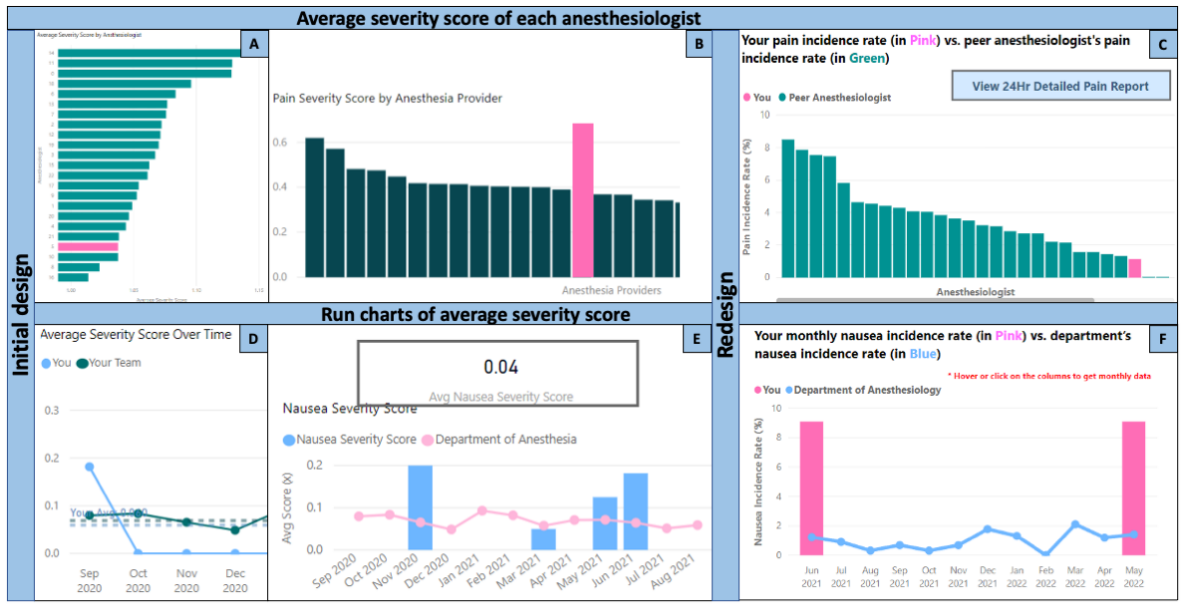
Progression of various sections of the dashboards with average severity scores in A, B, and pain incidence rates in C; monthly run charts of the anesthesiologist, along with team’s aggregate outcomes in D, E, and F.

Guided by previous reports [19,24-26], 2 sections were developed initially: (1) average severity scores of patient outcomes [24,26], and (2) run charts of average severity score (Figures 3A and 3D). Inspired by Parks et al [24], we initially designed a bar chart that displayed the anesthesiologists’ average severity scores in descending order, labeled with QIQA identifiers and the active anesthesiologist (user) highlighted (Figure 3A). Following feedback, we removed their codes to add a deidentification layer and added the severity score next to the bars (Figure 3B). The usability survey respondents indicated that the “pain severity score” calculation was unclear. Subsequently, we changed the metric from “pain severity score” (average of pain score categories) to “pain incidence rate” (occurrence of moderate or severe pain), which was more readily comprehensible (Figure 3C).

Based on co-design input, the active anesthesiologist’s average severity score and the department’s aggregate were initially represented in a monthly run chart with a median score superimposed (Figure 3D). The anesthesiologists subsequently suggested a run chart for the department-level score and a bar chart for the active anesthesiologist’s score (Figure 3E). Finally, the title was modified to specify that the plot now reported incidence rates, and the colors were modified for consistency with other plots.

#### PACU Outcome Summary

The second phase of development added PACU outcome summary to the dashboards. PACU nurse interventions requiring opioid (both ≥1 dose and ≥4 doses) or antiemetic (≥1 dose) administration were plotted. The progression in design was similar to the 24-hour outcome dashboard. Monthly average PACU opioid or antiemetic administration rates of both the active anesthesiologist and the department were included in the final dashboards.

#### Anesthesiologists’ Practice Profile

A practice profile page was designed based on anesthesiologists’ interest in incorporating individual case mix information (age category, sex, and surgical service) into their interpretation of their outcome data.

### Data Analysis

We summarized the 24-hour outcome data and PACU outcome data in the POFU database from the initial implementation of the data collection tool to illustrate the data available to anesthesiologists for visualization. We analyzed the anesthesiologists’ usability survey responses and presented the results descriptively, along with the final dashboard designs. Finally, we conducted a preliminary analysis of the impact of the dashboards on postoperative patient outcomes by comparing the baseline preimplementation period for PACU outcomes (April 2021 to June 2021) and 24-hour outcomes (September 2020 to June 2021) with the postimplementation period (September 2021 to February 2023) by plotting the month-by-month department incidence of each outcome and the overall average incidence for the period; to reduce bias, July 2021 to August 2021 have been plotted, but not considered in the comparison, as dashboard co-design was conducted during this period and was available to some members of the department. Changes between pre- and postimplementation periods were compared using the Wilcoxon rank-sum test.

## Results

### Data Set Characteristics

The 24-hour outcome dashboard contains data collected from September 2020 to February 2023. Of 12,082 total cases, 7877 (65.2%) postoperative phone calls were successfully completed to collect 24-hour outcomes from the family; 316 (4.0%) had moderate or severe pain, 73 (0.9%) had moderate or severe nausea, and 84 (1.1%) had moderate or severe vomiting. The distributions of age, sex, and PACU outcomes did not differ between patients with successful calls and those without. The PACU outcome dashboard contains 8716 cases collected from April 2021 to February 2023; 634 (7.3%) of these patients were administered at least 1 opioid dose, 78 (0.9%) required repeated opioid (≥4) doses, and 93 (1.1%) required an antiemetic.

### Usability Survey

The January 2022 usability survey (Multimedia Appendix 1) was completed by 17 of 29 (59%) anesthesiologists, including 1 of 17 (6%) who had been practicing 6-11 years, 5 of 17 (29%) practicing 11-15 years, 6 of 17 (35%) practicing 16-20 years, and 5 of 17 (29%) practicing >20 years; respondents included 4 of 17 (24%) who performed cardiac anesthesia as part of their practice, 6 of 17 (35%) who performed anesthesia for neurosurgery, and 4 of 17 (24%) who performed anesthesia for spine surgery. Among all respondents, 15 of 17 had used the dashboard during the 4 months since the initial version had been deployed, though only 2 of 17 (12%) had used it regularly. Of those who had used the dashboard, 9 of 15 (60%) reported that it was easy to navigate, and 9 of 15 (60%) thought the information was clearly presented. On the other hand, 3 of 15 (20%) users had found the navigation difficult, 2 of 15 (13%) thought the information needed to be clearer, and only 7 of 15 (47%) had found the help text and user instructions helpful.

Overall, the information provided was considered helpful by 12 of 15 (80%) of those that had used the dashboard, some of whom indicated that it had impacted their practice: for 8 of 15 (53%), this impact had been minimal, but 2 of 15 (13%) considered its impact significant. Comments indicated that the perceived benefits were primarily related to an increased awareness of the need for higher intraoperative analgesic and antiemetic dosing in some cases and an opportunity to engage with trainees on this issue. Most respondents (12/17, 71%) confirmed they were comfortable with how this information about their practice was being collected and presented to them, although 4 of 17 (24%) were concerned there may be negative consequences to having these data available. Concerns were that the quality indicators presented did not consider the multiple other influences on patient outcomes and, depending on how the indicators are subsequently used, could apportion blame inappropriately, with possible professional or legal consequences; this further highlights the need to guarantee provider confidentiality.

Concerning future use, 14 of 17 (82%) respondents recommended a regular reminder email, and 11 of 17 (64%) indicated they would refer to the dashboard at least monthly, with 10 of 17 (59%) believing that it had the potential to have a significant benefit for their practice. Additional information requirements identified for future work included: duration of PACU stay, 13 of 17 (76%); antibiotics timing, 8 of 17 (47%); perioperative hypothermia, 8 of 17 (47%); difficult intubation recorded, 6 of 17 (35%); and incidence of hypotension, 5 of 17 (29%).

### Final Dashboards Deployed

#### Overview

**Figure 4:**
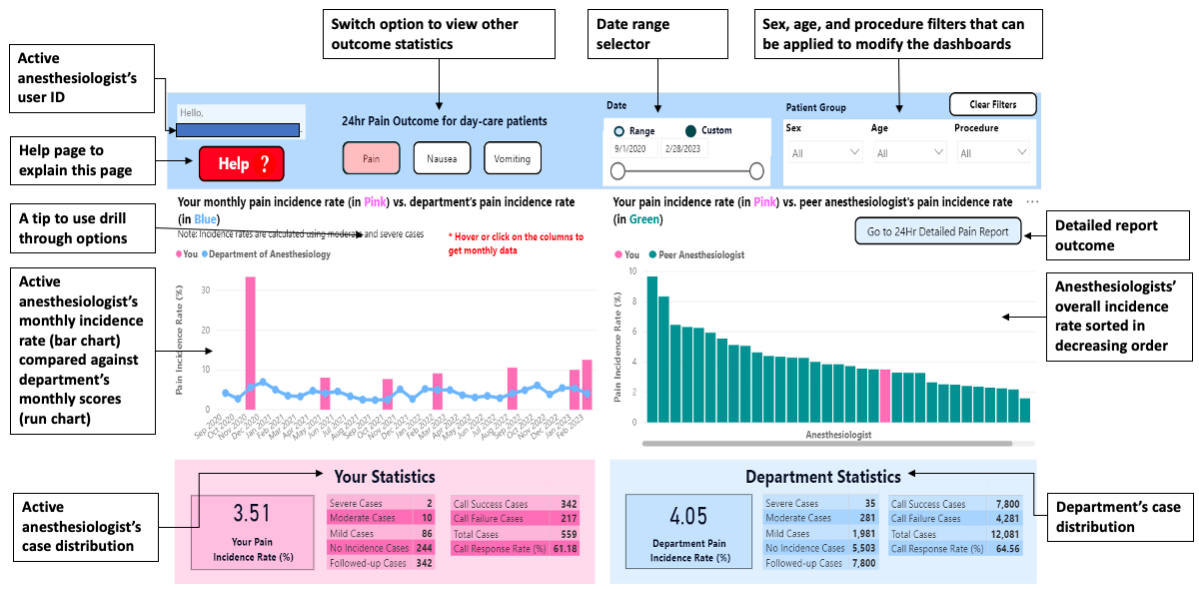
The final 24-hour postoperative outcome summary dashboard with notes.

**Figure 5:**
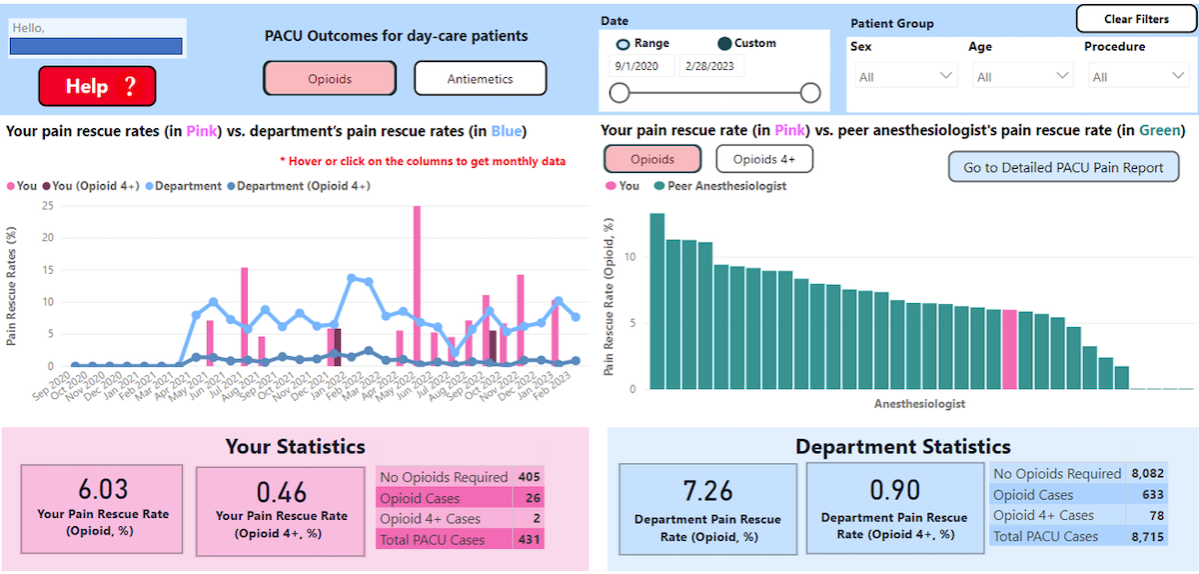
The final PACU outcomes dashboard design. PACU: postanesthetic care unit.

**Figure 6:**
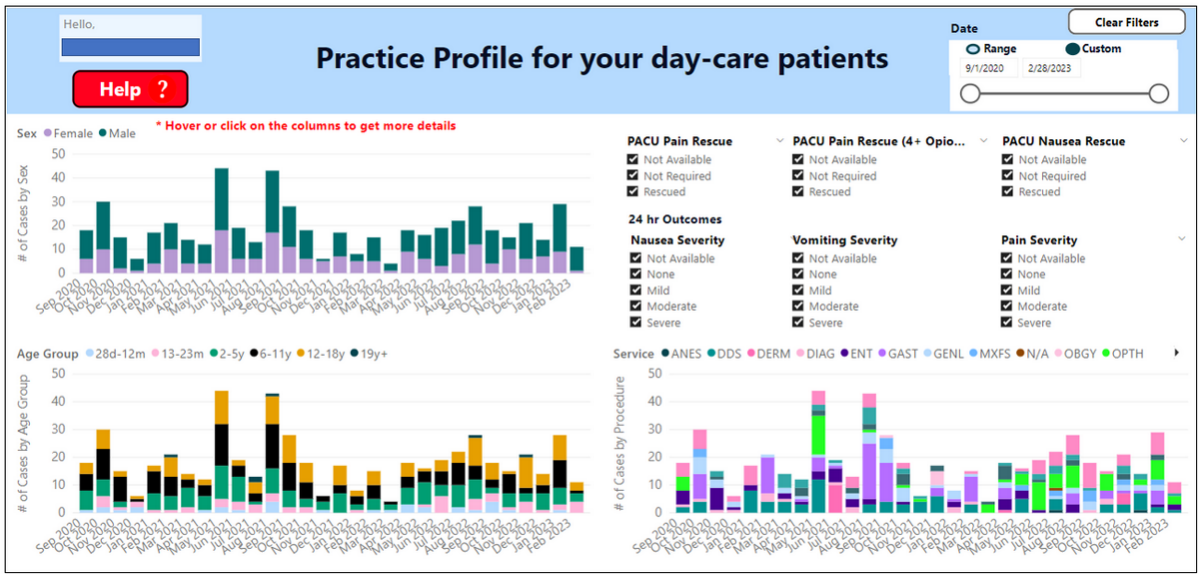
The final anesthesiologist practice profile dashboard design.

The final dashboard components, deployed in June 2022, are (1) 24-hour outcome summary (Figure 4), (2) PACU outcome summary (Figure 5), and (3) anesthesiologists’ practice profile (Figure 6).

#### 24-Hour Outcome Summary

The 24-hour outcome summary displays each outcome (pain, nausea, and vomiting) and compares the active anesthesiologist’s monthly incidence rate with the department’s rate (Figure 4, left). Their peers’ incidence rates are given in descending order, with the active anesthesiologist highlighted (Figure 4, right). Guided by the usability survey, we added the active anesthesiologist’s and overall department statistics (Figure 4, bottom).

The *View 24hr Outcome Report* button leads to a detailed report, where the anesthesiologist can view additional outcome information via switch tabs at the top of the page (Figure S1 in Multimedia Appendix 2): this report displays the anesthesiologist’s case distribution across the 4 severity levels and the outcome incidence rate by sex, age category, and procedure group. All data are shown compared to the department’s rates.

Both the outcome summary and the detailed report, support filtering based on patient sex, age category, and surgical service using drop-down menus. The “show data” option lets users view deidentified data contained within each plot. For a patient-level or filtered view of an anesthesiologist’s caseload, the user can apply a “drill-through” feature on all the personal charts to dive deeper into the data using advanced filters.

Based on the usability survey, we reconfigured the user instruction manual as a help page (Figure S2 in Multimedia Appendix 2), with a screenshot of the outcome page and information about each section, including formulas to calculate incidence rates.

#### PACU Outcome Summary

The PACU outcome summary uses the same visualization techniques as the 24-hour outcome summary: (1) plot for overall change over time and (2) for peer comparison, the opioid rescue rate is further divided into pain that required at least 1 dose and pain that required ≥4 doses of rescue medication (opioids 4+).

The PACU outcome summary shows opioid and antiemetic administration rates on separate pages: *PACU opioids* (Figure 5) and *PACU antiemetics* (Figure S3 in Multimedia Appendix 2). A toggle switch between opioids and opioids 4+ allows the anesthesiologists to view their performance against their peers (Figure 5, right). This PACU summary has the same help and data management capabilities as the 24-hour outcomes dashboard.

#### Final Anesthesiologists’ Practice Profile

A practice profile allows the anesthesiologist to view their case mix in stacked bar plots grouped by sex, age category, and surgical service in monthly intervals (Figure 6). The practice profile supports filtering the anesthesiologist’s case mix based on PACU and 24-hour postoperative outcomes. The user can also view the average outcomes of each caseload group as a tool tip or “drill-through” to see a detailed list of cases.

### Preliminary Analysis of Dashboard Impact

**Figure 7:**
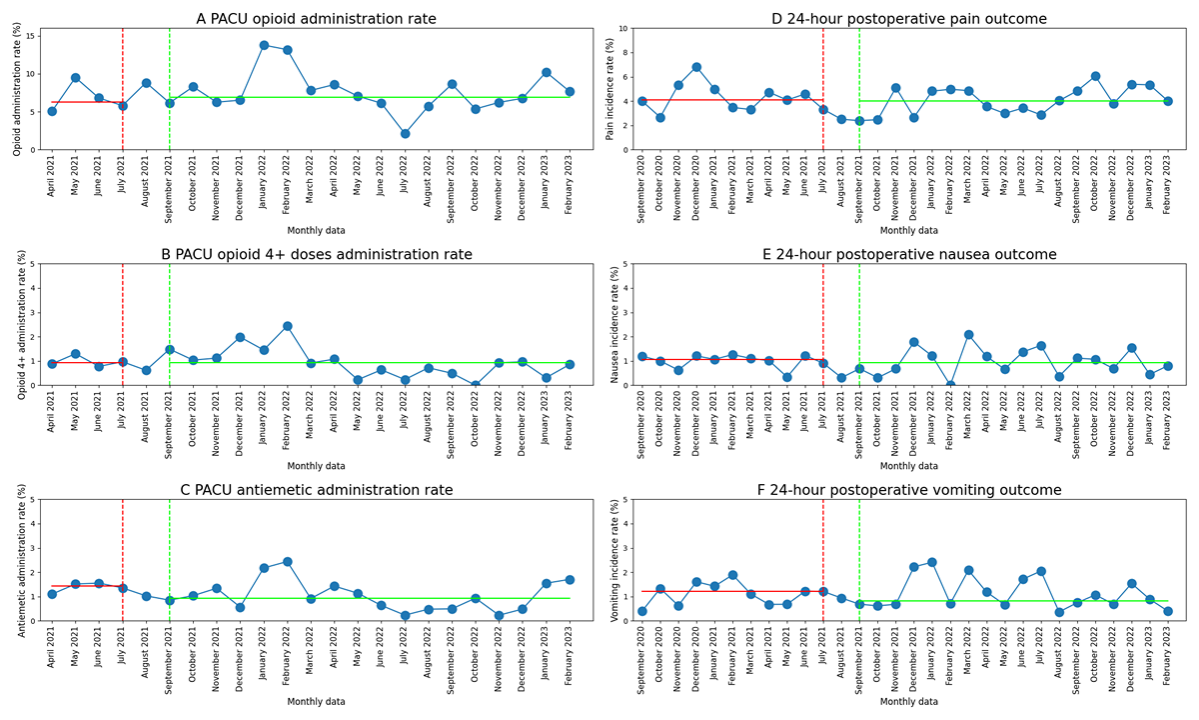
Variability in the department’s monthly aggregate PACU outcome rates, including (A) opioid rescue, (B) opioid rescue ≥4 doses, and (C) antiemetic administration, and 24-hour postoperative outcome rates, including (D) pain, (E) nausea, and (F) vomiting. The department’s monthly aggregate outcome rates are plotted as blue dots; the red dotted line indicates the end of the preimplementation period; the green dotted line indicates the start of the postimplementation period; the solid red and green horizontal lines indicate the medians of the pre- and postimplementation periods, respectively. PACU: postanesthetic care unit.

The department’s aggregate incidence rate for all outcomes is reasonably low but with significant month to month variability (Figure 7). Incidence rates were not different between the preimplementation and postimplementation periods: median differences were 0.7 (95% CI –2.5 to 3.4; *P*=.52) for PACU opioid administration, –0.0 (95% CI –0.7 to 0.5; *P*=.76) for PACU opioids 4+ administration, –0.5 (95% CI –1.0 to 0.2; *P*=.16) for PACU antiemetic administration, –0.1 (95% CI –1.2 to 0.8; *P*=.86) for 24-hour pain, –0.0 (95% CI –0.4 to 0.3; *P*=.88) for 24-hour nausea, and 0.0 (95% CI –0.5 to 0.5; *P*>.99) for 24-hour vomiting.

## Discussion

### Principal Results

We designed and implemented dynamic dashboards for the anesthesiologists in our department to review their outpatients’ postoperative short-term outcomes and compare these outcomes to their peers to facilitate personal performance evaluation and potential practice improvements. This project built on previous work establishing the POFU registry to collect and electronically aggregate these outcome data. The dashboard is updated weekly and presents (1) 24-hour outcomes, (2) PACU outcomes, and (3) a practice profile containing individual anesthesiologist’s case mix, grouped by age groups, sex, and surgical service. Co-design and usability evaluation indicated requirements for uncluttered summary data visualization and the ability to explore specific outcome details if needed. Anesthesiologists in our department generally found the dashboard information helpful and believed it would significantly benefit their practice. Our study confirmed that ensuring security and patient and provider confidentiality in these dashboards was crucial for successful uptake.

### Comparison With Prior Work

There are significant limitations in using postoperative outcomes such as pain, nausea, and vomiting as anesthesiologist performance indicators. There are many confounding factors, including procedural details and the surgeon being responsible for prescribing analgesics. These confounders will likely increase with increasing time after the procedure. However, even for immediate postanesthesia outcomes, confounders, including the PACU nursing team assessing and managing pain, have been shown to invalidate interanesthesiologist performance comparisons [27]. It is also essential to consider the impact of the case mix: Schulz et al [28] suggest that case-mix adjustment of measures such as length of PACU stay may provide more meaningful indicators than unadjusted metrics. However, the dashboard was not founded on the idea that an anesthesiologist’s practice is the sole determinant of their patients’ outcomes; it is a tool for anesthesiologists to reflect on their practice in context. Providing the data is only the first step toward better understanding their practices, but it is necessary. Anesthesiologists know that multiple variables outside their control contribute to these data, especially for 24-hour outcomes. Understanding their patients' variability should allow them to interpret this information appropriately.

Clinical dashboards are being widely explored in an attempt to better understand and optimize patient outcomes in a range of anesthesia settings [29], including cardiac [30] and pediatric anesthesia [18,20,31]. Data analytics focus on process, as well as outcome, metrics [32], and there is often a need to extract the required data from a range of in-hospital systems [31] or to supplement these data with patient- or family-reported outcomes [20,33]. These initiatives may or may not have a positive impact on performance. For example, regular team and individualized feedback reduced temperature monitoring delays during spine surgery among 1 group of anesthesiologists [7]. In contrast, another group found that audit and feedback did not improve the intraoperative temperature management [34]. However, such initiatives may often be the best route to improving practice, patient experience, and outcomes, particularly in acute pain management, given the challenges faced in implementing and adopting integrated electronic medical records in this area [35].

While dashboards and follow-up phone calls may function as intended, be economically justifiable, and be well received by anesthesiologists [20,31,36], it will be more challenging to demonstrate a beneficial impact on patient-relevant outcomes rather than process outcomes. Brenn et al [20] reported an 88% response rate for postoperative phone calls to 42,688 pediatric outpatients, but were unable to link satisfaction with complication rates and suggested that reducing wait times and streamlining operations are more important to families. In contrast, Kim et al [33] found that implementing a follow-up call (even from a nonmedical professional) 48 hours after day surgery reduced family anxiety, though it did not improve family satisfaction. We have yet to examine the effect of our dashboard on improving our patients' outcomes.

Through an iterative design process, we learned the importance of collaborating with the end users of the dashboards to maximize usability. This collaboration in the co-design process familiarized us with local clinical terminology (eg, “day-care patients” in preference to “ambulatory patients” or “outpatients”), which helped us design dashboards that are more locally relevant. These collaborative design processes have been adopted by other researcher-developers [18,19,23,31,33], although not all such dashboards have been co-designed with end users or undergone usability evaluation [17,30]. We used a usability survey to guide us toward more acceptable dynamic dashboards with various analysis options.

### Limitations

Some limitations of our dashboard designs and development approach should be noted. First, there is no risk adjustment of performance scores based on case mix: an anesthesiologist with a significant proportion of patients in whom postoperative pain or nausea have a higher baseline incidence cannot be validly compared to an anesthesiologist with a different case mix. The dashboard users recognize this limitation, and we implemented the practice profile section partly in an effort to address this issue. We may explore risk adjustment strategies in future but must be cautious not to overturn our users’ requirement for concise summary performance reports.

Second, the POFU registry includes age, sex, surgical service, and procedure date but does not contain other presurgical (risk) factors such as weight, comorbidities, existing medication, allergies, number or type of previous procedures, or child and parent anxiety levels. In the future, we plan to add additional patient data, surgical and anesthetic techniques, and PACU length of stay by integrating with the hospital’s Anesthesia Information Management System (SurgiNet Anesthesia, Cerner), which may allow us to provide our anesthesiologists with further insights.

Third, our current data are limited to outcomes occurring within 24 hours of discharge: PACU data recorded according to institutional practice and the 24-hour outcome data, for which a nurse makes only a single phone call to the patient’s family. The success rate to date has only been 7800 out of 12,081 (65%) cases; a higher rate would improve validity. To alleviate the missing 24-hour outcome data, we are exploring the development of self-reporting tools for pain, nausea, and vomiting, such as Panda [37], a mobile postoperative pain management app. We also aim to determine if families are willing to provide outcomes beyond 24 hours.

Finally, our usability questionnaire was not a standardized instrument or evaluated for reliability or validity, which limits its generalizability. It aimed to evaluate specific dashboard features as a final step before department-wide deployment.

### Further Evaluation

The dashboard version has been deployed and is being used by department anesthesiologists, with data updated weekly. We will perform an ongoing usability evaluation to examine the dashboards' usefulness, determine if anesthesiologists have any issues with the visualizations, and explore suggestions for additional information or functionality, including further optimization for use on a tablet or smartphone if required. We plan to evaluate usage patterns: how many anesthesiologists use the dashboards, how frequently, for what purpose, and over what period. Finally, we aim to extend our analysis of changes in the department’s aggregate and individual outcomes post deployment. Our preliminary analysis did not demonstrate any significant changes in outcomes, which may be in part because our PACU and 24-hour follow-up outcomes were already reasonably well-optimized compared to other institutions’ [38,39]. Our future work will focus on tracking and reducing the variability in outcome rates between different practitioners. Ultimately, evaluating the impact of this initiative on our patients’ outcomes is a significant undertaking and, hence, a long-term goal. It will involve integrating with our Anesthesia Information Management System (SurgiNet Anesthesia, Cerner), identifying other key outcomes that matter to our patients and their families, and applying the appropriate systems and resources to collect, process, and analyze this information.

### Conclusions

The dynamic, personalized dashboards we designed and deployed have allowed the anesthesiologists in our department to review their outpatients’ short-term postoperative outcomes and reflect on their practice in the context of peer and departmental performance. Key lessons from this implementation include the value of adopting a participatory approach to development, with co-design workshops and usability evaluation; the importance of establishing a robust approach to the security and confidentiality of patient and provider data in gaining user trust; and the preference for presenting uncluttered summary data combined with the opportunity to drill down into specific cases if required. This dashboard solution has allowed our department’s anesthesiologists to visualize previously unavailable data collected as part of a broader quality initiative. It should provide the opportunity to evaluate iterative practice changes, although further work will be required to monitor its effect on individual and department performance and patient outcomes.

## Appendix

Multimedia Appendix 1 POFU Anesthesia Dashboard - usability and utility survey.

Multimedia Appendix 2 View 24hr Outcome Report, Help page, and PACU Antiemetics.

## Abbreviations

BCCHR: British Columbia Children’s Hospital Research Institute
PACU: postanesthetic care unit
POFU: postoperative follow-up registry
QIQA: quality improvement or quality assurance
REDCap: Research Electronic Data Capture
