# Supplementary material for: Dashboard of Short-Term Postoperative Patient Outcomes for Anesthesiologists: Development and Preliminary Evaluation
Source: JMIR Perioper Med. 2023 Sep 19;6:e47398. doi: 10.2196/47398 (PMC10548316; doi:10.2196/47398)
Supplement: Multimedia Appendix 1 [file periop_v6i1e47398_app1.pdf]

# POFU Anesthesia Dashboard - usability and utility survey

The Pediatric Anesthesia Research Team would like to ask you confidential questions in this survey to assess the usability and utility of the recently implemented Post-Operative Follow-Up (POFU) anesthesia dashboard. The results of this survey will help us to learn more about how to improve its design and make it more tailored to your needs.

If you decide to complete the survey, you are consenting (agreeing) to participate. Completion of the survey is voluntary. It will take approximately 5-10 minutes to complete the survey.

Some of the questions in this survey relate to your professional information and others ask for your personal views/opinions. We hope that collecting this information will reveal useful information such as the relationships between demographic categories and perceptions of the dashboard.

Only the study team and the REDCap technical support team at the PHSA will access your information. The results of this survey are confidential. When results are reported, presented or published, the project team will remove any information that could identify you or anyone else. The team may use a quote from your comments if provided. Survey data will be stored securely at PHSA for 1 year.

Your personal information is protected by our privacy law in BC. This law is called the Freedom of Information and Protection of Privacy Act (FIPPA). We are collecting your information under section 26 (e) of FIPPA.

If you have questions about the survey or your information please contact: Matthias Görges, Rm V3-324, 950 West 28th Ave, Vancouver. BC V5Z 4H4; (604) 875-2000 x5616; mgorges@bcchr.ca

---

| | |
|---|---|
| I have read and understand this form.<br><br>I voluntarily consent to PHSA collecting, using, and disclosing the information I provide. | ◯ I consent<br>◯ I do not consent |

## Demographics

How many years have you been practising anesthesia?

- ◯ 0-5
- ◯ 6-10
- ◯ 11-15
- ◯ 16-20
- ◯ 21+

Do you practise any specific pediatric anesthesia sub-specialties?

(Please check all that apply)

- ☐ Cardiac
- ☐ Neuro
- ☐ Spine

## Your use of the anesthesia dashboard

| | |
|---|---|
| How often have you used the POFU anesthesia dashboard? | ◯ Never<br>◯ I have accessed it once or twice since September<br>◯ Monthly<br>◯ More frequently than monthly |
| Why have you not used the anesthesia dashboard? | ◯ I have not tried to access it<br>◯ I have tried but not been able to access it |
| Can you tell us more about the issue(s) you have encountered when trying to access the dashboard? | __________________________________ |
| Can you tell us why you have not accessed it (more frequently)? | __________________________________ |
| How often do you plan to look at the dashboard data in the future? | ◯ Never<br>◯ Irregularly (as needed)<br>◯ Monthly<br>◯ At least weekly |
| Can you tell us why you do not plan to use it (more frequently)? | __________________________________ |
| Would a reminder email to access the dashboard be useful? | ◯ No<br>◯ Yes, a monthly reminder<br>◯ Yes, a reminder every 2 weeks<br>◯ Yes, a weekly reminder |
| Please suggest any other way(s) you way you like to be reminded about the dashboard | __________________________________ |
| Would you be interested in looking at department's monthly average scores as an email? | ◯ Yes<br>◯ No |

## Ease of use

| | |
|---|---|
| How easy is the dashboard to navigate? | ◯ Very difficult<br>◯ Difficult<br>◯ Neither difficult nor easy<br>◯ Easy<br>◯ Very easy |
| Can you tell us more about the navigation issues you experienced? | __________________________________ |
| How clear is the information within the dashboard? | ◯ Very unclear<br>◯ Unclear<br>◯ Neither unclear nor clear<br>◯ Clear<br>◯ Very clear |
| Can you tell us more about what aspects you found unclear? | __________________________________ |
| Are the dashboard help text/user instructions helpful? | ◯ Very unhelpful<br>◯ Unhelpful<br>◯ Neither unhelpful nor helpful<br>◯ Helpful<br>◯ Very helpful |
| Can you tell us more about what is missing from the help text/user instructions? | __________________________________ |
| Do you know who to contact if you encounter problems with the dashboard? | ◯ Yes<br>◯ No |

Please contact Andrew Poznikoff at apoznikoff@bcchr.ca for more help.

## Dashboard content

| | |
|---|---|
| Is the information provided in the dashboards helpful? | ◯ Very unhelpful<br>◯ Unhelpful<br>◯ Neither unhelpful nor helpful<br>◯ Helpful<br>◯ Very helpful |
| Can you tell us more about what aspects were not helpful? | __________________________________ |
| Which of the following are useful pieces of information in the dashboard?<br><br>(please check all that apply) | ☐ PACU - Nausea & Vomiting<br>☐ PACU - Pain<br>☐ 24hr outcome - Nausea & Vomiting<br>☐ 24hr outcome - Pain |
| Why are these components important to you? | __________________________________ |
| Which of the following are not useful pieces of information in the dashboard?<br><br>(please check all that apply) | ☐ PACU - Nausea & Vomiting<br>☐ PACU - Pain<br>☐ 24hr outcome - Nausea & Vomiting<br>☐ 24hr outcome - Pain |
| Why are these components not important to you? | __________________________________ |
| In the future, which of the following pieces of information would be helpful in the dashboard?<br><br>(please check all that apply) | ☐ Antibiotics (ABX) timing<br>☐ Hypotension<br>☐ PEDI (Pediatric Difficult Intubation)<br>☐ DART (Difficult Airway Response Team)<br>☐ Duration of PACU stay<br>☐ Peri-operative hypothermia<br>☐ Other |
| What other information you would like to see in the dashboard? | __________________________________ |
| Do you have any suggestions for additional functionality in the dashboard design? | __________________________________ |

## Impact on your practice

| | |
|---|---|
| Has the information in the dashboard changed your practice? | ◯ Not at all<br>◯ Minimal change<br>◯ Significant change<br>◯ Very significant change |
| Did the information in the dashboard improve your practice? | ◯ Not at all<br>◯ Minimal improvement<br>◯ Significant improvement<br>◯ Very significant improvement |
| Can you tell us more about the change and/or improvement you have observed in your practice? | ______________________________ |
| Can you tell us more about why you think you have observed no change in your practice? | ______________________________ |
| Do you think the availability of the dashboard might benefit your practice in the future? | ◯ Not at all<br>◯ Minimal benefit<br>◯ Significant benefit<br>◯ Very significant benefit |
| Can you tell us why you think the dashboard will not benefit your practice going forward? | ______________________________ |

## Overall views on the POFU dashboard

| Question | Response |
| --- | --- |
| Are you comfortable with the way this information about your practice is collected and presented to you? | ◯ Very uncomfortable<br>◯ Uncomfortable<br>◯ Neither uncomfortable nor comfortable<br>◯ Comfortable<br>◯ Very comfortable |
| Can you tell us more about why you are not comfortable with capturing and presenting this information? | __________________________________ |
| Do you perceive any negative consequences to having this data available? | ◯ Yes<br>◯ No |
| Can you tell us more about the negative consequences you perceive to having these data available? | __________________________________ |
| Do you have any other feedback/comments on the dashboard? | __________________________________ |
| Would you be interested in providing one-on-one feedback?<br><br>(approximately 15mins) | ◯ Yes<br>◯ No |

Thank you - please contact Andrew Poznikoff at apoznikoff@bcchr.ca to arrange a feedback session
