# Supplementary material for: Dashboard of Short-Term Postoperative Patient Outcomes for Anesthesiologists: Development and Preliminary Evaluation
Source: JMIR Perioper Med. 2023 Sep 19;6:e47398. doi: 10.2196/47398 (PMC10548316; doi:10.2196/47398)
Supplement: Multimedia Appendix 2 [file periop_v6i1e47398_app2.pdf]

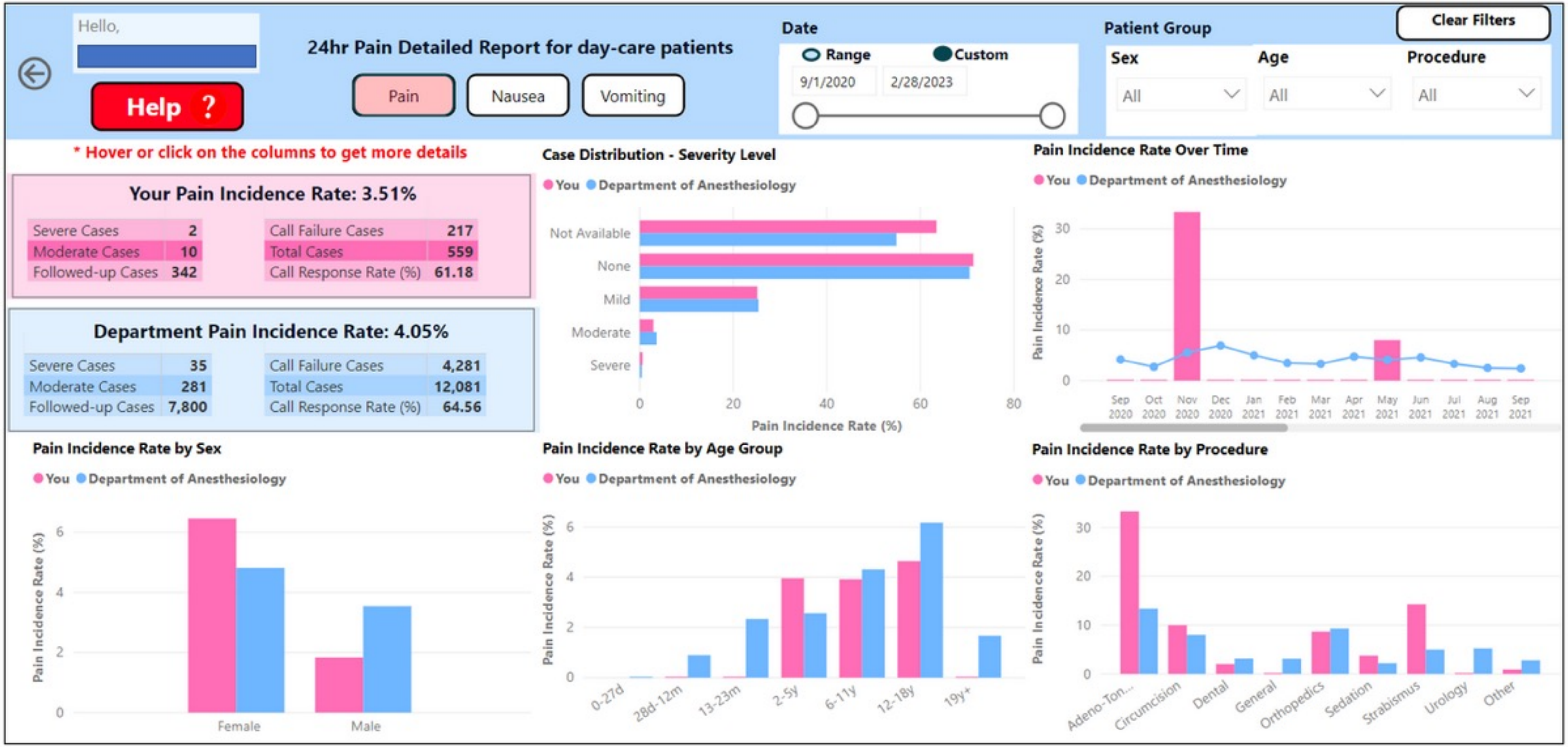

Figure S1: *24hr Outcome Report.* This is a detailed report, where the anesthesiologist can view additional outcome information via switch tabs at the top of the page. It displays the anesthesiologist's case distribution across the four severity levels and the outcome incidence rate by sex, age category, and procedure group. All data are shown compared to the department's rates.

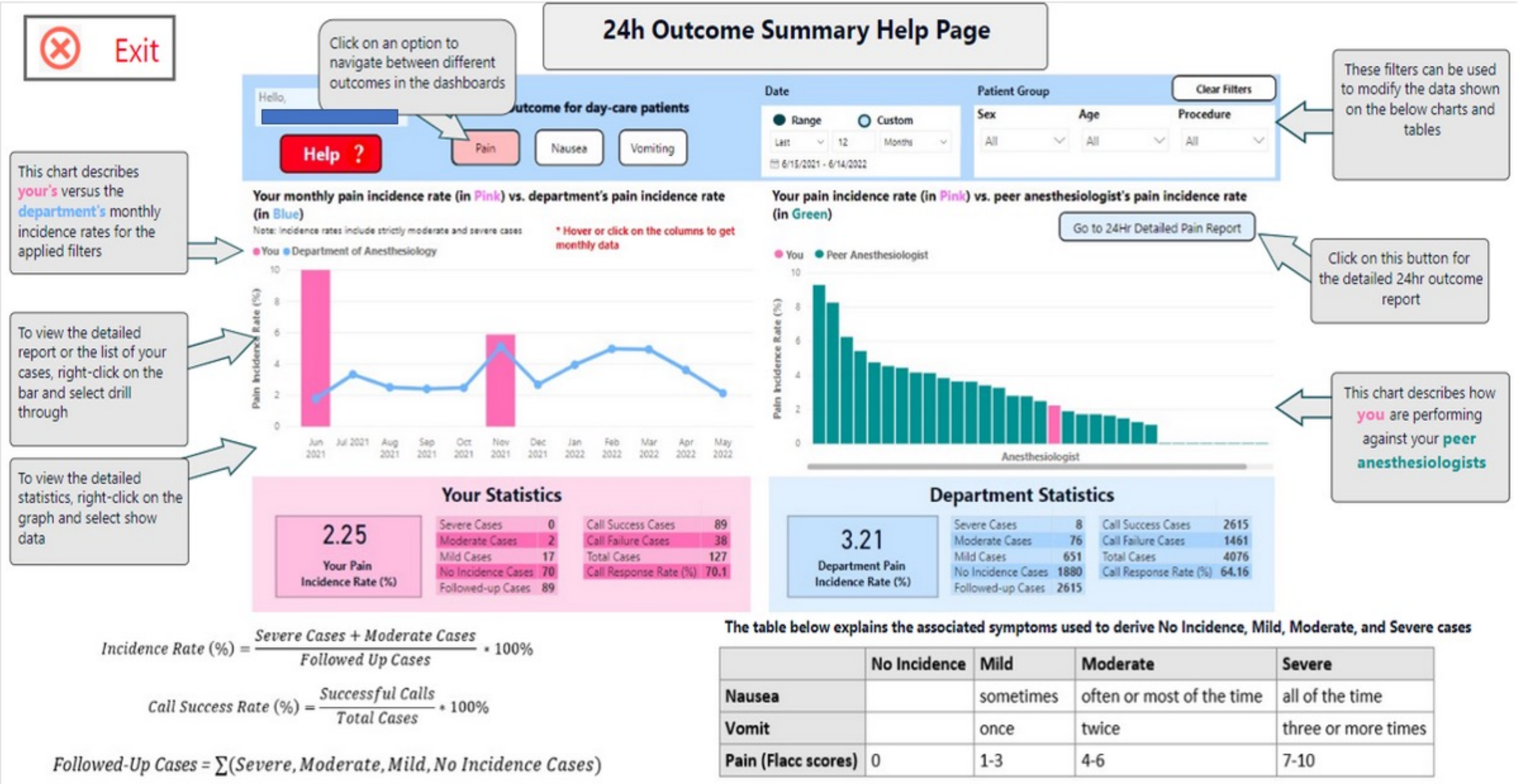

Figure S2: *Help page.* The user instruction manual is presented as a help page, with a screenshot of the outcome page and information about each section, including formulas to calculate incidence rates.

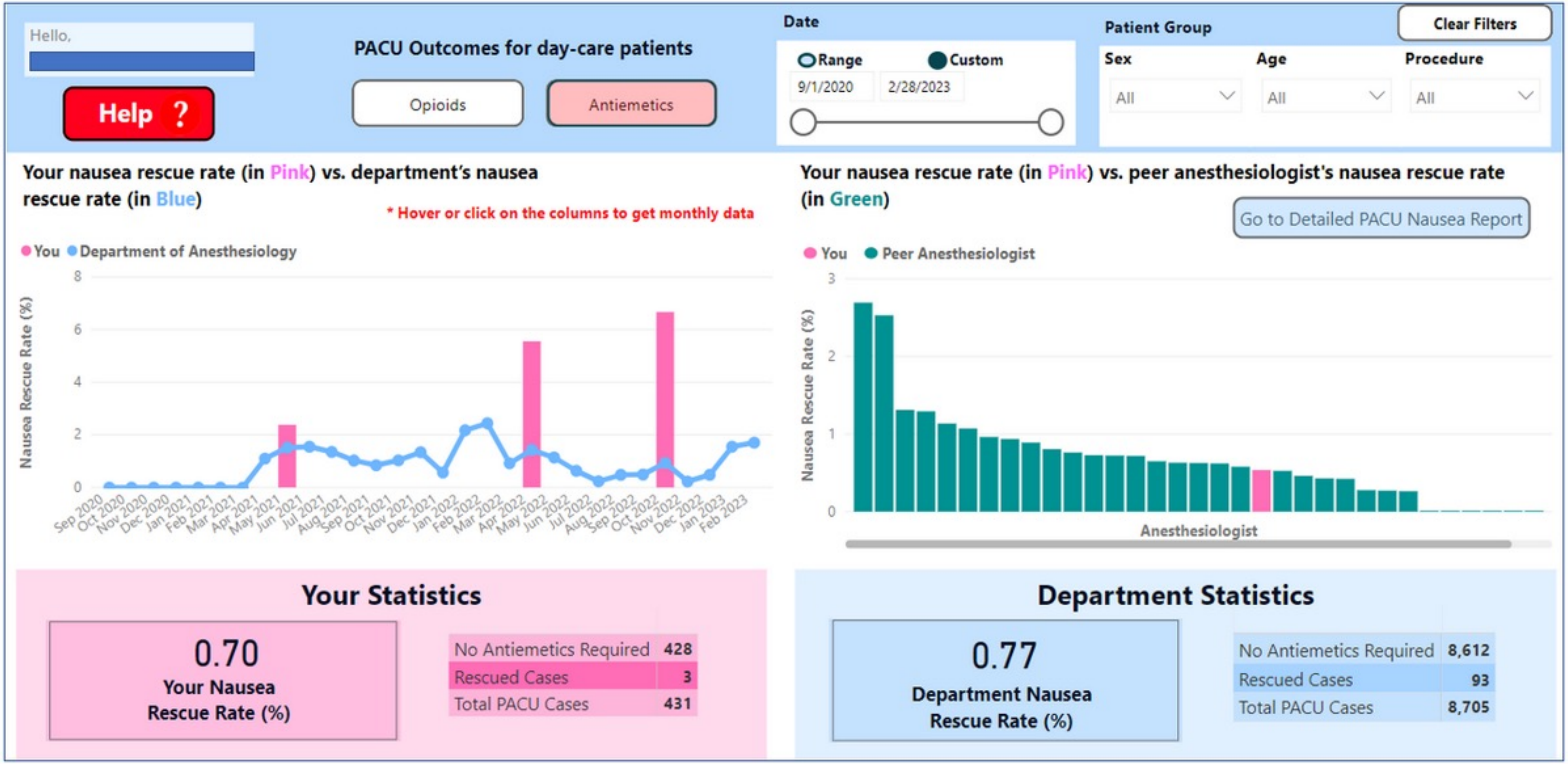

Figure S3: *PACU Antiemetics screen.* The PACU outcome summary shows opioid and antiemetic administration rates on separate pages.
